# Computer vision in precision livestock farming: artificial intelligence-driven technologies and applications for sustainable animal production

**DOI:** 10.5713/ab.260165

**Published:** 2026-03-24

**Authors:** Thi Thi Zin, Pyke Tin

**Affiliations:** 1 Graduate School of Engineering, University of Miyazaki, Miyazaki, Japan

**Keywords:** Animal Welfare, Artificial Intelligence, Computer Vision, Precision Livestock Farming

## Abstract

The growing global demand for animal-derived food products is placing unprecedented pressure on livestock production systems to improve efficiency while also assuring animal welfare, environmental sustainability and economic viability. Precision livestock farming (PLF) has emerged as a transformative paradigm that integrates advanced sensing technologies, computer vision, internet of things infrastructures and artificial intelligence (AI) to enable continuous, automated and individualized animal monitoring. This paper explores the evolution of livestock management from conventional observation-based practices to sophisticated, data-driven architecture. It also synthesizes recent advancements in PLF emphasizing its system architecture, key applications in cattle production, cross-sector expansion and emerging challenges. The core architecture of PLF is structured into three functional layers: (i) data acquisition through multi-modal sensors, with a primary emphasis in this review on visual and environmental monitoring system; (ii) data analytics employing machine learning and deep learning techniques to establish behavioral and physiological baselines; and (iii) decision-support mechanisms that translate analytics into actionable farm management interventions. Major applications, including individual animal identification, body condition score estimation, lameness detection, calving time prediction and AI-powered health monitoring, are critically discussed. The extension of PLF principles to aquaculture and other livestock sectors is also discussed. By shifting from herd-level to individual-animal management, PLF provides a scalable, non-invasive approach for early disease detection, optimized resource utilization, improved welfare standards and long-term economic sustainability. The current limitations, including high capital investment, data interoperability challenges and model generalizability constraints, have been analyzed and future research directions emphasizing explainable AI and welfare-oriented system design have been proposed. Overall, PLF represents a systemic transformation of animal agriculture, allowing for data-driven, sustainable and welfare-centered production systems.

## INTRODUCTION

**Figure 1:**
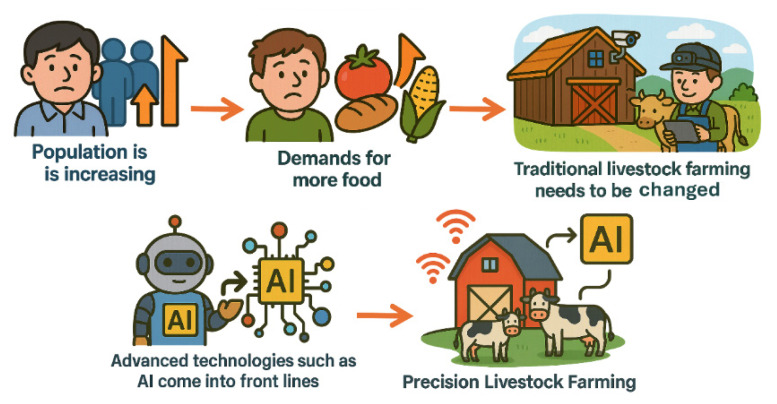
Evolution of livestock farming. AI, artificial intelligence.

Global food demand has been projected to increase significantly by 2050, driven by population growth, urbanization and evolving dietary preferences [1]. Livestock production systems must therefore intensify output while addressing growing societal concerns regarding animal welfare, environmental impact and food safety (Figure 1). This dual mandate of productivity and sustainability presents a complex challenge for modern animal agriculture.

Conventional livestock management relies heavily on periodic visual inspection, manual record-keeping and experiential knowledge. Although practical and widely adopted, such approaches are inherently subjective and often insufficient for detecting early-stage or sub-clinical health conditions. Delayed intervention can lead to compromised welfare, economic losses and increased disease transmission within herds.

Precision livestock farming (PLF) has emerged as a technological response to these limitations. By integrating real-time sensing systems with artificial intelligence (AI) and automated analytics, PLF enables continuous monitoring of individual animals rather than aggregate herd-level observations. This shift from reactive to predictive management marks a paradigm transition in livestock production [2–4].

PLF not only enhances health monitoring but also supports broader sustainability goals. Digital traceability strengthens transparency across the food value chain, while automation mitigates labor shortages in rural regions. When embedded within IoT-enabled infrastructures, PLF systems facilitate semi-autonomous environmental control and closed-loop management strategies. Nevertheless, technological, economic and ethical challenges must be addressed to ensure responsible implementation at scale [5,6]. This review provides a comprehensive examination of PLF technologies, applications in cattle production, benefits and limitations and future directions for sustainable animal agriculture.

## OVERVIEW OF PRECISION LIVESTOCK FARMING: DEFINITION AND OBJECTIVES

**Figure 2:**
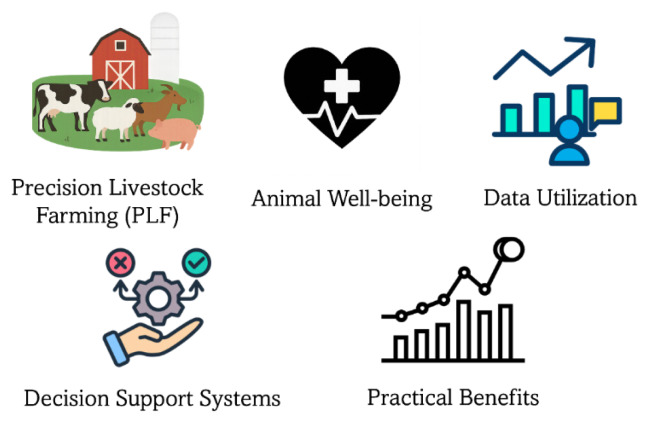
Overview of precision livestock farming.

PLF is the integration of advanced technologies to monitor, analyze and manage livestock in real time at the individual animal level (Figure 2). Unlike traditional herd management, PLF focuses on per-animal variables to optimize outcomes [7–9]. The primary objectives of PLE center on enhancing animal health and welfare through early intervention while simultaneously improving operational efficiency by optimizing feed conversion ratios and reducing veterinary costs. Furthermore, these systems support long-term sustainability by minimizing the environmental footprint of intensive farming and facilitating a transition toward evidence-based, data-driven management.

## ARCHITECTURE OF PRECISION LIVESTOCK FARMING SYSTEM

**Figure 3:**
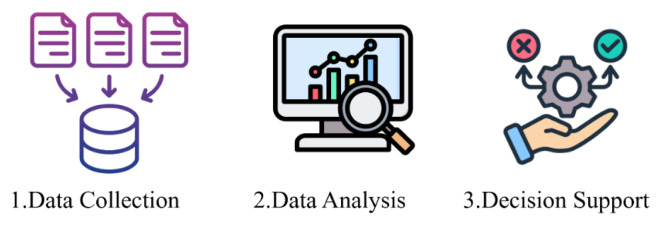
General architecture of precision livestock farming system.

The PLF systems operate through an integrated three-layer architecture: data acquisition, data analytics and decision support (Figure 3).

### Data acquisition layer

The data acquisition layer captures high-resolution, multimodal information from animals and their environment. Technologies include:

• 2D and 3D vision systems
• Depth cameras
• Wearable sensors (collars, ear tags and accelerometers)
• Environmental IoT sensors (temperature, humidity and gas concentration)

Animal-level data typically includes posture, gait, feeding behavior, rumination activity, locomotion patterns and body morphology. Environmental parameters such as barn climate, ventilation efficiency and air quality are parallelly monitored to contextualize behavioral responses. Continuous, non-invasive data collection forms the foundation for individualized digital phenotyping.

### Data analytics layer

Raw sensor data have been transmitted to centralized or cloud-based platforms where machine learning and deep learning models perform preprocessing, feature extraction and behavioral modeling.

This layer performs:

• Data cleaning and synchronization
• Baseline establishment for individual animals
• Anomaly detection through temporal modeling
• Predictive risk assessment

Advanced algorithms such as convolutional neural networks (CNN), recurrent neural networks (RNN), hidden Markov models (HMM) and regression-based frameworks enable extraction of meaningful health and welfare indicators from complex datasets.

### Decision support layer

The decision-support layer translates analytical outputs into actionable management interventions. Outputs may include:

• Real-time dashboard alerts
• Mobile notifications
• Automated actuation (ventilation, feeding systems and sorting gates)

**Figure 4:**
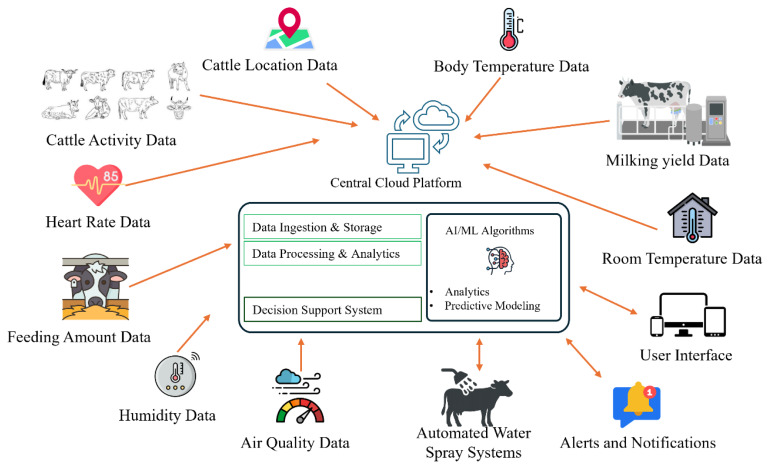
Combination of main three layers in precision livestock farming system. AI, artificial intelligence; ML, machine learning.

This layer operationalizes predictive insights into practical farm-level responses, reducing reliance on manual supervision and enabling timely intervention (Figure 4).

## KEY APPLICATIONS IN CATTLE PRODUCTION

### Individual identification and tracking

Accurate individual identification is foundational to PLF. Computer vision–based systems now enable non-invasive recognition of cattle using ear tags, facial features, coat patterns, or 3D morphological signatures.

Multi-object tracking algorithms combine spatial positioning, motion trajectory analysis and appearance-based descriptors to maintain identity consistency in high-density barn environments. Compared with radio frequency identification (RFID) or global position system (GPS) systems, image-based approaches reduce hardware costs and avoid signal interference while providing continuous visual auditing capability.

Reliable individual tracking facilitates personalized monitoring of feeding behavior, health progression and welfare indicators.

In this context, we have developed a specialized computer vision framework designed to track individual cows using ear tags as the primary visual identifier. This research aims to address the persistent challenge of identifying animals in crowded farm settings where traditional methods often fail. While GPS and RFID systems have been widely used, they can be cost-prohibitive or suffer from signal interference within metallic barn structures.

**Figure 5:**
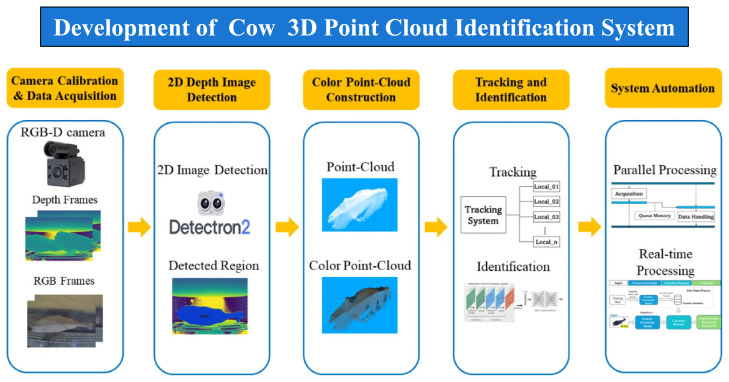
Overall architecture of cow 3D point cloud identification. Adapted from Kyaw et al [14] with CC-BY.

Our previous research has explored various methodologies to overcome the inherent limitations of traditional livestock monitoring by leveraging computer vision for non-invasive identification. Initial efforts focused on an image-based solution using standard surveillance cameras to recognize and track individual cows via ear tag data [10]. This was subsequently expanded through facial region analysis to facilitate both individual identification and the estimation of feeding durations [11]. To enhance operational robustness, we introduced an AI-driven system capable of real-time cattle tracking across diverse and challenging environmental conditions [12]. More recent advancements have utilized sophisticated deep learning architectures [13], including an automated approach that integrates color-point clouds through a hybrid PointNet++ Siamese network to achieve high-precision identification (Figure 5) [14].

Having established the framework for individual identification, the following section discusses the application of these technologies in behavioral monitoring.

### Automated body condition score estimation

**Figure 6:**
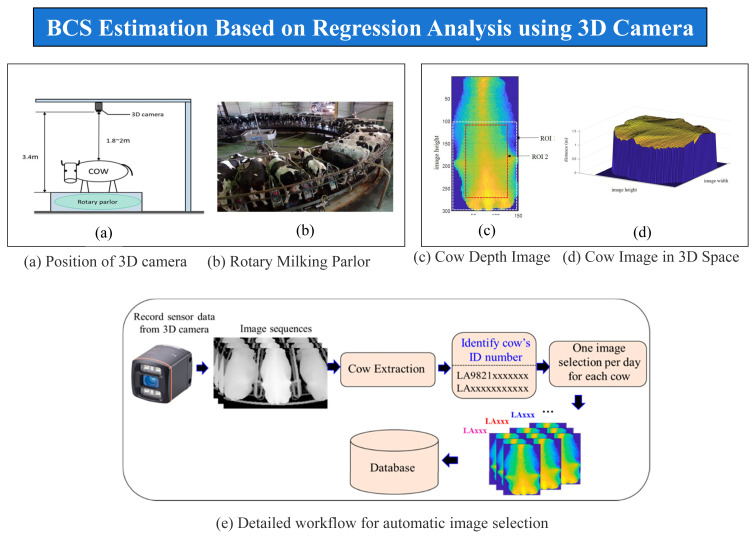
Overall architecture of cow body condition score (BCS) estimation. Adapted from Zin et al [15] with CC-BY.

Body condition score (BCS) is a critical indicator of metabolic status, reproductive readiness and production performance. Traditional scoring methods are subjective and exhibit inter-observer variability. PLF systems employ depth cameras and 3D imaging to capture morphological features along the backbone, hip and tail head regions. Extracted geometric features have been processed using regression models or deep learning frameworks to estimate BCS objectively (Figure 6).

Continuous longitudinal monitoring of BCS trajectories provides greater predictive value than single-point assessments, particularly during transition periods such as late gestation and early lactation. Automated BCS estimation enhances nutritional management and reduces metabolic disorders.

The BCS for cows indicates their energy reserves, the scoring for which ranges from very thin to overweight [15]. These measurements are especially useful during calving, as well as early lactation. Achieving a correct BCS helps avoid causing calving difficulties, losses and other health problems. Although BCS can be rated by experts, it is time-consuming and often inconsistent when performed by different experts [15]. In this context several researchers have studied from various perspectives. Among them, we have researched deeply using self-collected real-life data from small to large cattle farms located in the Miyazaki, Hokkaido and Oita prefectures in Japan. To outline a few, automatic evaluations of a cow’s body-condition score using 3D cameras have been introduced in [16], giving some promising results. In addition, [15] analyzed BCS estimation based on regression analysis using a 3D camera with some significant outputs.

### Lameness detection

Lameness represents a significant welfare and economic challenge in dairy production, characterized by altered gait patterns, abnormal posture and reduced mobility [17]. These physical manifestations often result in chronic pain, decreased milk productivity, reproductive issues and increased mortality rates [18]. Early and accurate detection is therefore crucial for prompt intervention, which mitigates long-term productivity losses and reduces treatment costs [19,20].

Traditional detection relies on manual locomotion scoring, which is labor-intensive, subjective and difficult to scale for large herds. To address these limitations, we propose an automated computer vision-based framework utilizing depth image analysis. Unlike standard RGB feeds, depth imaging allows for the precise extraction of 3D morphological features, such as spine curvature, stride variability and weight-shifting behavior, while remaining resilient to the inconsistent lighting conditions typical of barn environments.

**Figure 7:**
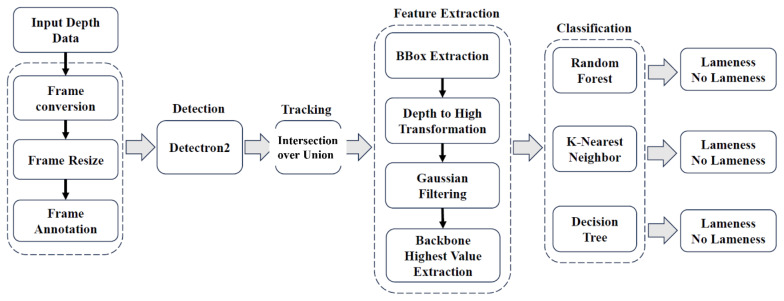
Overall pipeline of the proposed cow lameness classification system. Adapted from Tun et al [21] with CC-BY.

Our specific approach advances the field by focusing on the following technical components (Figure 7):

• Multi-cow tracking: We implement multi-cow detection and segmentation, utilizing intersection over union (IoU) analysis to maintain individual identities across frames.
• Feature extraction: We target the highest points along the bovine backbone, extracting specific feature vectors from the depth data to quantify spinal arching, which is a primary indicator of lameness.
• Classification: These extracted features serve as inputs for three distinct machine learning classifiers, which categorize the severity of lameness automatically.

By providing continuous, real-time monitoring, this holistic system eliminates observer bias and enhances the scalability of health monitoring in large-scale dairy operations [21–23].

### Calving time prediction

Calving is a critical physiological event characterized by high stress and significant mortality risks. Predicting the exact onset of labor is essential for providing timely assistance and reducing complications related to dystocia, defined as prolonged, abnormal, or difficult birth [24]. Dystocia is a primary concern for dairy producers, as it leads to substantial economic losses, high labor demands and a neonatal mortality rate that can reach 35% internationally [25,26]. Consequently, effective management of the calving process has been considered a priority in dairy research to prevent long-term illness and improve survival rates [27].

**Figure 8:**
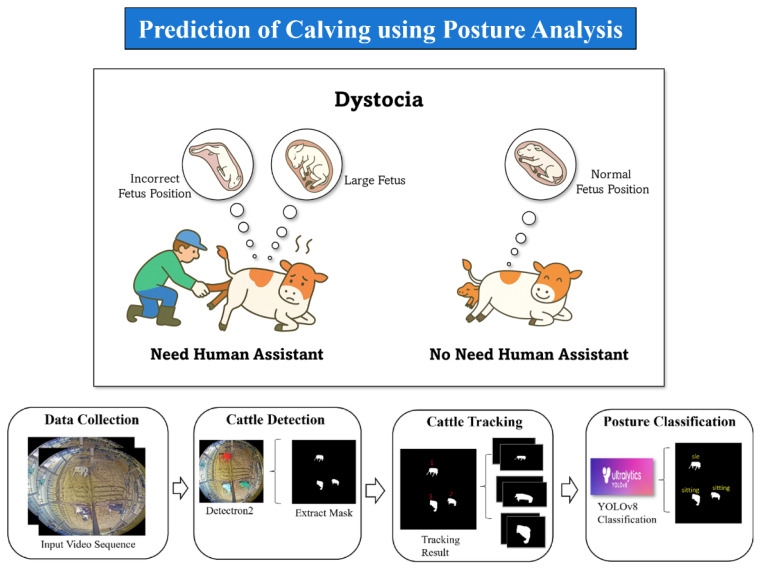
Cow posture analysis system architecture: abnormal and normal cow calving. Adapted from Khin et al [31] with CC-BY.

PLF systems address these challenges by providing continuous, non-invasive video monitoring. These systems utilize time-series modeling to identify pre-parturition behavioral signatures that are often too subtle for intermittent human observation. Key indicators (Figure 8) identified for calving prediction include significant shifts in posture transitions, such as changes in lying and standing frequency, alongside increased tail activity and specific positioning. These behavioral trajectories provide critical data for distinguishing between normal pre-parturition behavior and the active onset of labor.

**Figure 9:**
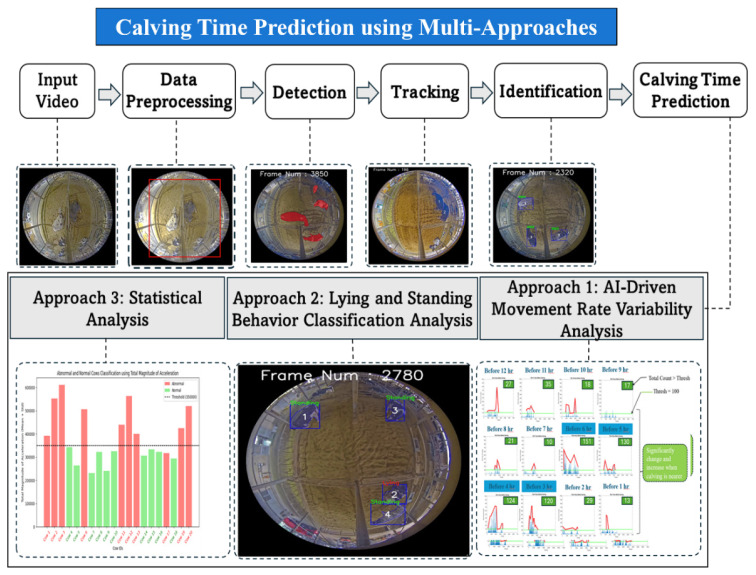
Cow calving time prediction using multiple approaches [34]. Adapted from Mg et al [34] with CC-BY.

In our research, we have developed a precise video-monitoring framework that distinguishes between normal pre-calving behavior and the active onset of labor. Our methodology (Figure 9) combines behavioral data extracted from recorded sequences with advanced probabilistic frameworks, including HMM and Absorbing Markov Chain Models, alongside various machine learning techniques [28–30] and deep learning techniques [31–34]. By automating these alerts, the system minimizes the need for 24-hour human surveillance while ensuring that farm personnel can intervene at the optimal moment, thereby enhancing both animal welfare and operational sustainability.

### Artificial intelligence-powered health monitoring

Modern precision agriculture is currently shifting toward holistic “e-health” frameworks, which utilize integrated e-monitoring systems to assess general welfare indicators non-invasively. These systems represent a significant advancement over traditional livestock monitoring, such as manual inspection and physical handling, which are often labor-intensive, subjective and stressful for the animals. To address these limitations, we have developed an AI-powered visual e-monitoring system designed to enhance cattle health management and overall farm productivity [35].

The core of this system is a structured deep learning pipeline that processes continuous visual data from farm-integrated surveillance cameras. This automated solution functions through three primary stages:

• Data processing: Video streams undergo preprocessing and feature extraction to isolate key behavioral markers.
• Behavioral analysis: By analyzing posture, movement patterns and visible physical abnormalities, the system identifies subtle deviations from baseline behavior that may indicate the early onset of illness or distress.
• Real-time intervention: When abnormal patterns are detected, the system generates immediate alerts, enabling farmers to provide timely treatment before a condition escalates.

**Figure 10:**
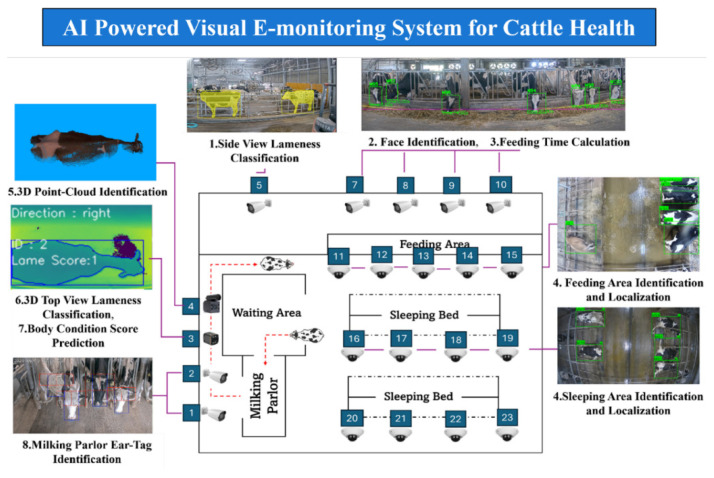
Overall cow health monitoring system and camera installation. Adapted from Moe et al [35] with CC-BY.

Our research demonstrates that integrating AI into livestock monitoring significantly reduces reliance on manual observation while enhancing the accuracy and speed of health assessments. By detecting diseases in their prodromal stages, farmers can minimize productivity losses and optimize resource allocation. These AI-driven systems (Figure 10) provide a practical, scalable solution for modern precision agriculture, contributing to improved animal welfare, operational efficiency and long-term profitability [35].

## BENEFITS AND CHALLENGES

### Benefits of precision livestock farming

PLF offers substantial advantages that transcend traditional management practices by shifting toward predictive and individualized animal care. A primary benefit is the capacity for early sub-clinical disease detection, which allows for medical intervention before clinical symptoms manifest. This proactive approach is complemented by improved feed efficiency and resource optimization, which simultaneously reduces labor dependency and operational costs. Furthermore, PLF frameworks provide quantifiable welfare indicators and enhanced traceability, fostering greater transparency within the supply chain. Collectively, these technological integrations facilitate increased productivity and animal longevity, directly contributing to the sustainable intensification of livestock production.

### Challenges and limitations

Despite its transformative potential, several systemic barriers remain that hinder the widespread adoption of PLF technologies. High initial capital investment remains a significant hurdle for many producers, often compounded by the technical complexity of data interoperability and the integration of disparate sensing systems. From a modeling perspective, the limited generalizability of trained algorithms across different farm environments poses a challenge, as models developed in specific geographical or management contexts frequently require extensive adaptation before broader deployment. Moreover, the transition to digital management introduces concerns regarding data privacy and ownership, alongside a critical requirement for enhanced farmer training and digital literacy. Finally, ethical considerations surrounding automated decision-making must be addressed to ensure that algorithmic outputs align with both animal welfare standards and human oversight.

Algorithmic models developed in one geographical or management context may require adaptation before broader deployment. Additionally, ethical considerations surrounding automated decision-making must be addressed.

## FUTURE PERSPECTIVES

### Explainable and welfare-oriented artificial intelligence

Future PLF systems must incorporate Explainable AI frameworks to enhance farmer trust. Transparent algorithms that provide interpretable reasoning behind alerts will strengthen adoption and facilitate collaboration between technology and veterinary expertise.

Human-in-the-loop models, where automated analytics support rather than replace professional judgment, represent a balanced pathway forward.

### Cross-sector expansion: aquaculture

The PLF principles have increasingly been applied in aquaculture, where computer vision systems quantify shoaling behavior, feeding response and stress indicators. Transitioning from qualitative observation to data-driven monitoring enhances welfare assessment and feeding optimization.

Digital transformation in aquaculture mirrors developments in terrestrial livestock systems, indicating the broader applicability of precision farming technologies.

## CONCLUSIONS

PLF represents a systemic transformation of animal agriculture. By integrating non-invasive sensing technologies with advanced analytics and automated decision-support systems, PLF enables predictive, individualized and welfare-centered livestock management.

Although financial, technical and ethical challenges remain, continued interdisciplinary collaboration among engineers, animal scientists, veterinarians and producers will be essential to realize the full potential of PLF. When responsibly implemented, PLF offers a viable pathway toward sustainable, transparent and humane animal production systems capable of meeting future global food demands.

## Data Availability

Upon reasonable request, the datasets of this study can be available from the corresponding author.
